# Beyond the Air–Bone Gap: The Role of Bone Conduction Thresholds in Predicting Functional Outcomes and Guiding Surgical Decision-Making in Active Middle Ear and Bone Conduction Implants

**DOI:** 10.3390/audiolres16020046

**Published:** 2026-03-17

**Authors:** Joan Lorente-Piera, Raquel Manrique-Huarte, Sebastián Picciafuoco, Janaina P. Lima, Valeria Serra, Manuel Manrique

**Affiliations:** Department of Otolaryngology, Clinica Universidad de Navarra, 31008 Pamplona, Spainjpatriciode@unav.es (J.P.L.); mmanrique@unav.es (M.M.)

**Keywords:** bone conduction implants, active middle ear implants, chronic otitis media, Vibrant Soundbridge, cholesteatoma, bone conduction thresholds

## Abstract

**Introduction**: In patients with conductive and mixed hearing loss, implantable hearing devices such as active middle ear implants (AMEIs) and bone conduction implants (BCIs) are established alternatives when conventional hearing aids fail. Although bone conduction (BC) thresholds are routinely used as eligibility criteria, their role as frequency-specific predictors of postoperative functional outcomes remains poorly defined. This study aimed to evaluate the influence of preoperative BC thresholds across the audiometric spectrum on postoperative speech recognition outcomes after implantation with AMEIs and BCIs. **Methods**: A retrospective observational study was conducted at a tertiary referral center including patients implanted with BCIs or AMEIs. Pre- and postoperative audiological data were analyzed, including air and bone conduction thresholds, frequency-segmented BC measures (low, mid, and high frequencies), cochlear frequency gradient (ΔBC Slope), and speech recognition scores (SRSs) at 65 dB HL one year after implantation. **Results**: 102 patients were included (50 BCI, 52 AMEI). Both implant types achieved significant postoperative improvements in tonal thresholds and SRS compared with pre-implantation values (all *p* < 0.001). High-frequency BC thresholds (BC-High, 4–6 kHz) showed a significant inverse correlation with postoperative SRS in both BCI (r = −0.382, *p* = 0.001) and AMEI users (r = −0.398, *p* < 0.001), and emerged as the only independent predictor in multivariable models (BCI: β = −0.533, *p* = 0.022; AMEI: β = −0.491, *p* = 0.020). Low- and mid-frequency BC measures were not associated with postoperative speech outcomes (all *p* > 0.05). ROC analyses demonstrated excellent discriminative performance of BC-High for identifying suboptimal outcomes, with area under the curve values of 0.92 for BCI (*p* = 0.001) and 0.94 for AMEI (*p* = 0.002), and implant-specific cutoff values of >47 dB HL and >61 dB HL, respectively. **Conclusions**: High-frequency BC thresholds showed the strongest association with postoperative speech recognition after implantable hearing rehabilitation. BC-High could function as a prognostic marker of functional outcome rather than an eligibility criterion, providing clinically meaningful information to refine preoperative counseling and individualized decision-making within current indication frameworks.

## 1. Introduction

Hearing rehabilitation in patients with conductive and mixed hearing loss has evolved substantially over the past two decades, driven by the limitations of conventional hearing aids and the refinement of implantable auditory technologies. Many patients with chronic otitis media (COM), ossicular chain disruption, congenital malformations, external auditory canal atresia, or intolerance to conventional amplification fail to achieve stable and satisfactory functional benefit with hearing aids, particularly in anatomically complex ears [1,2]. In this setting, implantable devices have become key tools to restore auditory function.

Active middle ear implants (AMEIs) and bone conduction implants (BCIs) are the two most widely adopted implantable alternatives. AMEIs are semi-implantable devices that deliver mechanical vibration to the ossicular chain or directly to the inner ear via the oval or round window, bypassing dysfunctional middle ear mechanics [3]. The Vibrant Soundbridge (VSB) is the most widely used AMEI and has shown consistent audiological and functional benefit in sensorineural, mixed, and conductive hearing loss. Initially indicated for sensorineural hearing loss through incus coupling [4], its indications have expanded to include COM, cholesteatoma surgery, failed otosclerosis procedures, and congenital middle/external ear malformations [3,5]. Long-term series, including reports from our group, describe stable improvements in pure-tone thresholds and speech discrimination, with particularly relevant high-frequency gains when round-window coupling is employed [6].

BCIs transmit sound through skull vibration, bypassing the external and middle ear, and are widely used in conductive and mixed hearing loss as well as selected cases of single-sided deafness (SSD) [7]. Current systems include passive percutaneous, passive transcutaneous, and active transcutaneous designs. Although percutaneous devices historically provided efficient transmission, they carry higher rates of soft-tissue complications and implant loss [8,9,10]. Meta-analytic evidence adjusted for follow-up duration indicates lower minor-complication rates for active transcutaneous devices, whereas passive percutaneous systems show the highest incidence of major adverse events and revision surgery, supporting a shift toward fully transcutaneous active solutions [11]. In pediatric populations, BCIs remain particularly relevant for congenital conductive loss and aural atresia, though prospective data show that implant loss remains higher than in adults, consistent with the biological vulnerability of the growing temporal bone and soft tissues [12,13].

Unlike BCIs, AMEIs avoid skin-related complications and osseointegration constraints, but they require more demanding surgery and precise vibroplasty coupling, with coupling efficiency and middle ear status being key determinants of outcome [14]. Nevertheless, AMEIs have demonstrated durable long-term performance and high user compliance, even in chronically operated ears [6], and recent evidence suggests that advanced age alone does not preclude benefit [15].

Despite the extensive clinical use of both technologies, direct comparative data remain limited and are often heterogeneous in design and outcome selection. Recent work from our group comparing passive middle ear reconstruction with implantable solutions in COM reported superior audiological gains and lower reintervention rates for both AMEIs and BCIs, with AMEIs achieving greater speech and frequency-specific amplification and BCIs providing a favorable balance between benefit and surgical morbidity [16]. However, a persistent clinical challenge is identifying which patients are most likely to achieve meaningful functional benefit and which may be better served by alternative strategies, including cochlear implantation, particularly in borderline cases. Many underlying otological conditions are associated with progressive or longstanding deterioration of bone conduction (BC) thresholds, reflecting variable cochlear involvement. Consequently, decision-making often occurs in scenarios where middle ear pathology coexists with limited cochlear reserve.

Although BC thresholds are routinely incorporated into candidacy criteria, they have largely been treated as rigid eligibility cutoffs rather than explored as continuous, frequency-dependent predictors of postoperative functional outcome. We hypothesized that postoperative speech outcomes after implantable rehabilitation are shaped by the level and configuration of preoperative cochlear reserve, potentially modulated by clinical factors and by the selected implant strategy.

The present study aims to investigate the role of preoperative BC across the audiometric frequency spectrum as a determinant of functional outcomes one year after implantation with BCIs and AMEIs in patients with conductive, mixed, and sensorineural hearing loss. Specifically, we sought to assess whether the degree and frequency distribution of preoperative cochlear reserve, as reflected by BC thresholds, influences postoperative speech perception outcomes and to evaluate its value as a decision-making parameter when selecting among implantable hearing rehabilitation strategies. Secondary objectives include examining the association between frequency-segmented and global preoperative BC measures and postoperative functional performance, comparing outcome patterns between implant types across different levels of cochlear reserve, identifying clinical and audiological factors associated with favorable functional results. Even more, we also explored clinically relevant BC thresholds that may assist in patient selection in borderline cases between implantable middle ear or bone conduction devices.

## 2. Materials and Methods

### 2.1. Study Design

A retrospective observational study was conducted at a tertiary care center, following the ethical guidelines of the 1975 Declaration of Helsinki. Written informed consent was obtained from all participants.

### 2.2. Inclusion Criteria

**Figure 1:**
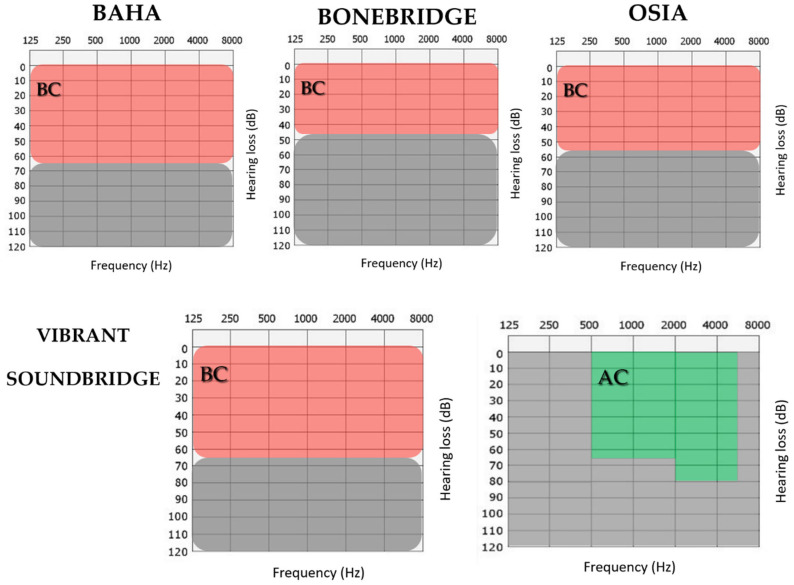
Audiometric indication ranges for implantable hearing devices. The (**upper**) row shows BCI indication ranges, with red shaded areas indicating the accepted bone conduction (BC) threshold ranges across frequencies. The (**lower**) row corresponds to the VSB, with red shading representing BC eligibility criteria and green shading indicating the air conduction (AC) threshold indication range. Gray areas denote audiometric thresholds outside the recommended indication ranges.

Pre- and postoperative data were collected from patients who underwent implantation with bone conduction devices (BCIs) (Baha^®^ Connect, Cochlear Ltd., Sydney, Australia; Bonebridge^®^, MED-EL, Innsbruck, Austria; or Osia^®^ OSI200, Cochlear Ltd., Sydney, Australia) or active middle ear implants (AMEIs), specifically the Vibrant Soundbridge VORP503 (MED-EL, Innsbruck, Austria). The audiometric profiles varied by implant type and are represented in Figure 1:

- Bone conduction implants [17]: No retrocochlear or central hearing disorders, with realistic expectations and better speech understanding possible with amplification. For the different devices available:
  ○ Baha Connect: Conductive or mixed hearing loss with bone conduction PTA ≤ 65 dB at 0.5, 1, 2, 3, and 4 KHz using the SuperPower sound processor.
  ○ Bonebridge: Patients older than 5 years with bone conduction thresholds not exceeding 45 dB HL.
  ○ Osia: Patients with bone conduction thresholds not exceeding 55 dB HL.
- Active middle ear implant (Vibrant Soundbridge). Eligible patients were older than 5 years and presented conductive, mixed, or sensorineural hearing loss without retrocochlear or central auditory disorders, with realistic expectations and better speech understanding possible with amplification [18]:
  ○ Air conduction PTA thresholds between 40 and 65 dB at 500 Hz, up to 80 dB at 6000 Hz.
  ○ Bone conduction thresholds compatible with sufficient cochlear reserve, typically not exceeding 65 dB HL at 2000 and 3000 Hz.
  ○ Speech recognition scores (SRS) ≥ 50% at 65 dB in open-set word test.
  ○ Audiometric inadequacy with other devices, or repeated dermatitis/external otitis contraindicating hearing aids.

### 2.3. Exclusion Criteria

Patients implanted with bone conduction devices for single-sided deafness (SSD) solely to mitigate the head-shadow effect were excluded, as this indication does not reflect the rehabilitative context addressed in the present study. Patients who had not completed the 1-year postoperative follow-up at the time of analysis were also excluded. Additional exclusion criteria included poor adherence to the implanted device, significant psychiatric comorbidities potentially compromising informed consent, data reliability, or compliance with the study protocol, as well as refusal or inability to provide written informed consent or to participate in the study.

### 2.4. Surgical Procedure

Bone conduction implant surgeries, both percutaneous and transcutaneous, were typically performed under local anesthesia and sedation, with linear flaps for BAHA and Bonebridge and U or italic S-shaped flaps for Osia. The mastoid area was drilled to fit the implant, depending on the surgeon’s preference.

AMEI implantation involved either ossicular chain attachment (incus or stapes) or round- or oval-window approaches. The standard procedure involved mastoidectomy and posterior tympanotomy for FMT anchoring, performed under general anesthesia in all cases.

When the underlying cause of hearing loss was chronic otitis media (COM), with or without cholesteatoma, surgical management included either a canal wall down mastoidectomy, with or without external auditory canal closure, or more conservative approaches such as the on-demand technique for cholesteatoma, known as attic exposure and antroexclusion (AE-AE) [19]. The choice of surgical strategy was based on disease extent, as well as patient- and surgeon-related considerations.

### 2.5. Follow-Up, Audiological and Clinical Variables

Postoperative follow-up included medical visits at 1 week, 4 weeks (implant activation), 3 months, 6 months, and 1 year, with continuous monitoring of the otological condition. Audiometric data were collected pre-implantation and compared with free-field results obtained at 1 year post-implantation. Key variables for auditory performance comparison included pre- and post-implant pure-tone thresholds and gains in both pathways—bone conduction and air conduction—measured in decibels at 250, 500, 1000, 2000, 4000, and 6000 Hz (AC40 audiometer, Interacoustics AS, Assens, Denmark). Specifically, the spectral distribution of cochlear reserve was characterized using frequency-segmented BC measures. Low-frequency BC (BC-Low) was calculated as the mean of thresholds at 250 and 500 Hz, mid-frequency BC (BC-Mid) as the mean of thresholds at 1000 and 2000 Hz, and high-frequency BC (BC-High) as the mean of thresholds at 4000 and 6000 Hz. The cochlear frequency gradient (ΔBC Slope) was defined as the difference between high- and low-frequency BC values following the next formula:

ΔBC Slope=ΔBC High−ΔBC Low

This simplified metric (ΔBC Slope) was included as an exploratory parameter to reflect the predominance of high-frequency cochlear deterioration relative to preserved low-frequency thresholds. Although these frequency-segmented measures do not correspond to conventional PTA definitions, they were intentionally used to capture both the level and configuration of cochlear reserve across the audiometric spectrum and to explore their relationship with postoperative speech recognition beyond global threshold averages. Speech recognition scores (SRSs) were assessed pre- and post-implantation using the Cárdenas and Marrero disyllabic word test, presented in free-field conditions at 65 dB SPL [20].

Finally, clinical variables such as age, sex, side of implantation, duration of hearing loss (in years), type of hearing loss (conductive, sensorineural or mixed), etiology, implant type, and, in case of AMEIs, the anchoring (round and oval window, incus or stapes) were also included.

### 2.6. Statistical Analysis

Continuous variables were assessed for normality using the Shapiro–Wilk test. Data results are presented as mean ± standard deviation (SD). Baseline comparisons between implant groups (BCI vs. AMEI) were performed using the Mann–Whitney U test for continuous variables and the Chi-square test or Fisher’s exact test for categorical variables. Within-group pre- and postoperative comparisons of SRS were conducted using paired t tests or Wilcoxon signed-rank tests, depending on data distribution.

Correlations between postoperative SRS and preoperative audiological variables were initially explored using Spearman’s rank correlation coefficients. These analyses were performed separately for each implant type and focused on frequency-segmented bone conduction measures (BC-Low, BC-Mid, BC-High) as well as the cochlear frequency gradient (BC Slope). To evaluate the independent contribution of preoperative audiological and clinical variables to postoperative outcomes, multivariable linear regression models were constructed separately for each implant group, using postoperative SRS at 65 dB as the dependent variable. Covariates included BC-Low, BC-Mid, BC-High, age at implantation, duration of hearing loss, etiology, and surgical technique (canal wall down or AE–AE). Regression coefficients (β) with 95% confidence intervals (CI) are reported. To ensure model stability relative to the sample size and maintain interpretability, a parsimonious modeling strategy was adopted, restricting the regression to the most clinically relevant audiological and demographic variables.

For clinically oriented analyses, receiver operating characteristic (ROC) curve analyses were performed to assess the ability of preoperative high-frequency bone conduction thresholds (BC-High, 4000–6000 Hz) to discriminate postoperative functional outcomes. For this purpose, postoperative speech recognition was dichotomized, defining suboptimal functional outcome as an SRS < 50% at 65 dB HL. The area under the ROC curve (AUC) was calculated to quantify discriminative performance, and optimal cutoff values were identified using the Youden index. Sensitivity and specificity were reported for clinically relevant thresholds.

When multiple comparisons were performed, *p*-values were adjusted using Bonferroni correction as appropriate. Statistical significance was set at a two-sided alpha level of 0.05. All statistical analyses were conducted using GraphPad Prism 10.4.0 (GraphPad Software Inc., San Diego, CA, USA).

## 3. Results

### 3.1. Study Population

A total of 124 patients were initially eligible. Of these, 18 patients implanted with BCIs for single-sided SSD to address the head-shadow effect were excluded, as this indication falls outside the scope of the present study. In addition, four patients with AMEIs had not yet completed the 1-year follow-up and were therefore excluded from the outcome analysis. Consequently, 102 patients were finally included in the study. All included patients who were indicated for an implantable device were users with good adherence to the device in question. Of these, 50 patients (49.02%) were BCI users, while the remaining 52 patients (50.98%) were VSB users. Demographic characteristics, clinical and etiological variables, and between-group differences are summarized in Table 1.

Overall, the comparative analysis between BCIs and active middle ear implants AMEIs revealed several statistically significant differences across key clinical variables. Patients receiving AMEIs were significantly older at the time of implantation than those treated with BCIs (*p* = 0.002). In addition, the distribution of hearing loss type differed markedly between groups (*p* < 0.001), with BCIs being predominantly indicated for conductive and mixed hearing loss, whereas AMEIs were more frequently used in patients with mixed and sensorineural hearing loss. Etiological profiles also showed significant intergroup differences (*p* < 0.001), reflecting distinct underlying disease patterns associated with each implant type. By contrast, no significant differences were observed in sex distribution, ear laterality, or duration of hearing loss evolution. Within the BCI group, among the 22 patients with transcutaneous devices, 16 were users of the Bonebridge system (32.00%) and 6 of the Osia device (12.00%).

Regarding etiological classification, COM refers to sequelae of non-cholesteatomatous chronic otitis media; stenosis denotes external auditory canal obliteration of either idiopathic or iatrogenic origin, and malformations include a spectrum ranging from atresia auris to idiopathic middle ear anomalies or syndromic conditions such as Treacher Collins syndrome (*n* = 3). Genetic cases were non-syndromic, and presbycusis referred to patients unable to use hearing aids due to chronic infectious conditions of the external auditory canal.

**Figure 2:**
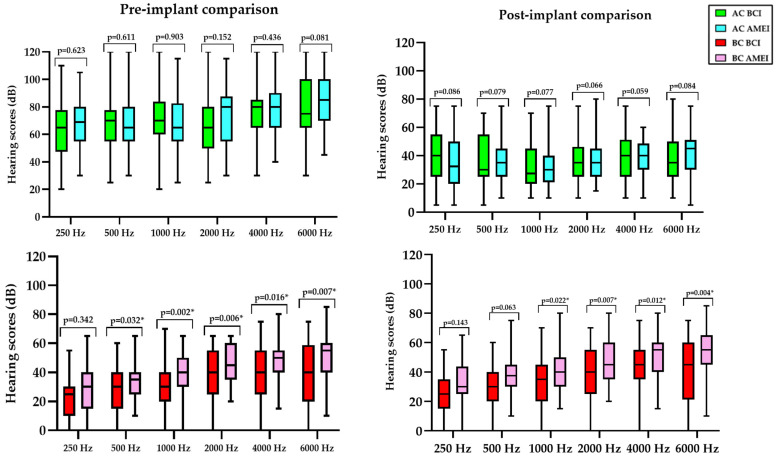
Frequency-specific AC and BC thresholds before and after implantation in patients treated with BCIs and AMEIs. The (**upper**) panels display AC thresholds across frequencies from 250 to 6000 Hz, whereas the (**lower**) panels depict pre- and post-implant BC thresholds. * *p* < 0.05.

Table 2 and Figure 2 summarize the audiological profile and surgical outcomes of patients treated with BCIs and AMEIs, including AC thresholds, BC thresholds, mean PTA, and SRS at 65 dB. At baseline, no statistically significant differences were observed between groups across AC or BC thresholds at any tested frequency, as illustrated in the pre-implant boxplots, with all frequency-specific intergroup comparisons yielding non-significant *p*-values. Likewise, global audiometric measures confirmed comparable preoperative status between groups, with no differences in mean PTA (Mann–Whitney U test, *p* = 0.233) or SRS at 65 dB (*p* = 0.281).

Within-group comparisons of pre-implant versus post-implant performance revealed a clear and consistent auditory benefit for both implant types. For all tested frequencies, Wilcoxon signed-rank analyses demonstrated statistically significant improvements when comparing baseline and postoperative conditions (*p* < 0.001). AC thresholds showed substantial postoperative gains in both groups, resulting in comparable tonal outcomes, as reflected by the absence of intergroup differences in postoperative mean PTA (*p* = 0.272). In contrast, postoperative free-field SRSs differed significantly between devices, with higher SRSs in the AMEI group (*p* = 0.024), indicating a differential impact of implant type on suprathreshold auditory performance.

Importantly, BC results showed a distinct and clinically meaningful pattern. Across most frequencies, BC thresholds were poorer in the AMEI group compared with the BCI group, with several frequency-specific comparisons reaching statistical significance. This finding is concordant with the underlying etiological and audiometric profiles of the cohorts: AMEI candidates predominantly presented mixed hearing loss patterns with a sensorineural component, whereas BCI recipients were mainly affected by purely conductive losses, as previously described in Table 1.

### 3.2. Influence of Preoperative BC Thresholds and Postoperative Speech Recognition Outcomes

**Figure 3:**
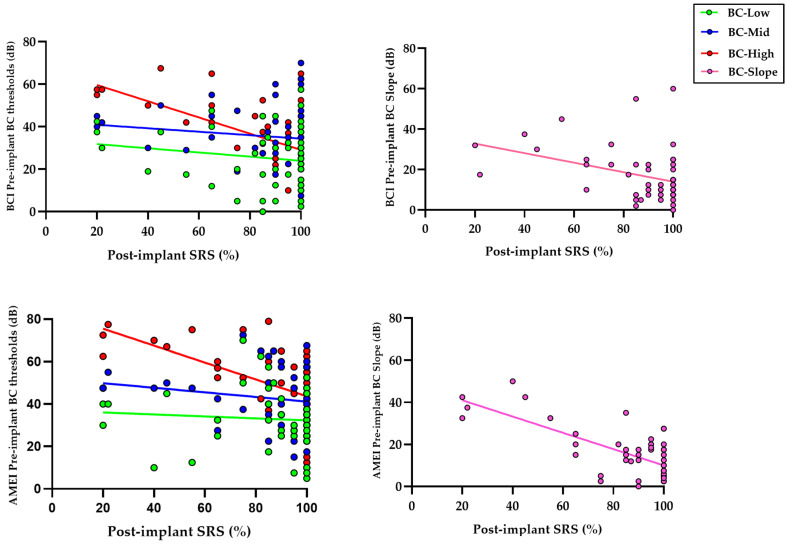
Correlation between postoperative SRS and preoperative frequency-segmented BC thresholds (BC-Low, BC-Mid, BC-High) and cochlear frequency gradient (BC Slope) in BCI and AMEI users.

As shown in Table 3 and Figure 3, the correlation between preoperative BC thresholds and postoperative SRS was assessed using Spearman’s rank correlation analysis, separately for BCI and AMEI users. BC-High (4000–6000 Hz) showed a significant inverse association with postoperative SRS in both implant groups, with correlation coefficients indicating a moderate relationship (BCI: r = −0.382, *p* = 0.001; AMEI: r= −0.398, *p* < 0.001). These values suggest that although high-frequency cochlear reserve contributes meaningfully to postoperative speech outcomes, it explains only part of the overall variability in functional performance. In contrast, low-frequency (BCI-Low, 250–500 Hz) and mid-frequency BC (BC-Mid, 1000–2000 Hz) did not show significant associations with postoperative SRS in either group.

Beyond absolute thresholds, the cochlear frequency gradient (BC Slope) also demonstrated a significant inverse association with postoperative speech recognition in both BCI (r = −0.329, *p* = 0.017) and AMEI users (r = −0.503, *p* = 0.002). Higher BC Slope values, reflecting a predominance of high-frequency cochlear deterioration relative to low frequencies, were associated with poorer speech outcomes, particularly among AMEI users. These findings suggest that while absolute high-frequency BC thresholds represent the primary determinant of postoperative speech recognition, the spectral distribution of cochlear reserve, as captured by this parameter, provides additional prognostic information.

### 3.3. Clinical and Audiological Factors Associated with Postoperative Speech Recognition Outcomes

Table 4 summarizes the results of the multivariable linear regression analyses performed separately for (BCI) and (AMEI) users. In both models, high-frequency BC thresholds (BC-High, 4000–6000 Hz) emerged as a significant independent predictor of postoperative speech recognition. Specifically, worse BC-High was associated with lower SRS outcomes in both BCI (β = −0.533, 95% CI −0.983 to −0.082; *p* = 0.022) and AMEI users (β = −0.491, 95% CI −0.901 to −0.081; *p* = 0.020). These findings indicate that the degree of cochlear reserve in the high-frequency range consistently conditions functional speech performance after implantation, independently of other clinical and surgical variables included in the models.

Beyond BC-High, age showed a differential effect between implant strategies. While age was not associated with postoperative SRS in BCI users (*p* = 0.938), it emerged as an independent negative predictor in the AMEI group (β = −0.454, 95% CI −0.869 to −0.039; *p* = 0.033). In contrast, BC-Low, BC-Mid, disease evolution, etiology, and surgical techniques did not demonstrate independent associations with postoperative SRS in either group.

The overall multivariable model reached statistical significance for AMEI users (overall model *p* = 0.008), indicating a meaningful combined explanatory value of the included predictors. By comparison, the overall model for BCI users did not reach statistical significance (overall model *p* = 0.131), reflecting the predominance of BC-High, an important determinant of functional outcome in this subgroup.

### 3.4. ROC Analysis of High-Frequency Bone Conduction Thresholds According to Implant Type

**Figure 4:**
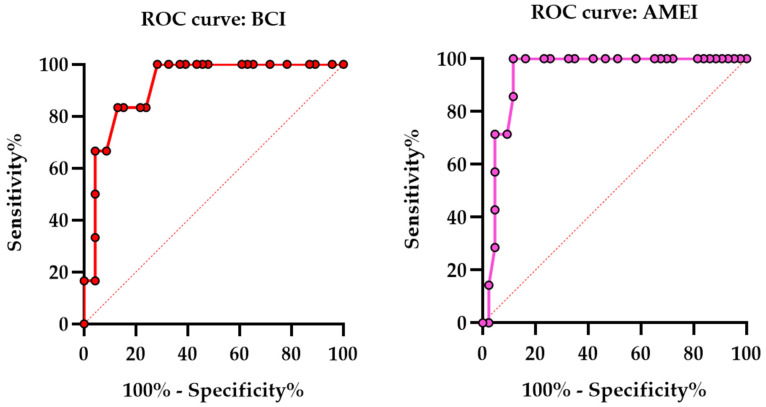
ROC curves illustrating the discriminative performance of BCIs ((**left**) panel) and AMEIs ((**right**) panel).

As illustrated in Figure 4, ROC analyses were conducted to evaluate the ability of high-frequency BC thresholds (BC-High, 4–6 kHz) to discriminate postoperative suboptimal speech recognition outcomes (SRS < 50%) separately in patients treated with BCIs and AMEIs.

In the BCI group, BC-High demonstrated strong discriminative performance for identifying patients at risk of suboptimal speech recognition (AUC = 0.9167, *p* = 0.001). The ROC curve showed a steep initial ascent followed by a broad plateau, indicating high sensitivity across a wide range of BC-High values and suggesting a relatively broad functional tolerance to high-frequency cochlear deterioration in BCI recipients. Using the Youden index, an exploratory cutoff value of >47.00 dB HL was identified, yielding 100% sensitivity and 71.74% specificity.

In the AMEI group, BC-High showed similarly strong discriminative capacity (AUC = 0.9402, *p* = 0.002), with an ROC curve displaying a sharper transition between favorable and suboptimal outcomes. This steeper profile suggests a more abrupt decline in functional performance once a critical level of high-frequency cochlear deterioration is exceeded. The maximum Youden index was observed at an exploratory cutoff of >61.00 dB HL, providing 100% sensitivity and 88.37% specificity. These thresholds should be interpreted as exploratory indicators of risk, derived from the present dataset, rather than definitive clinical decision limits.

## 4. Discussion

The present study shows that BC-High (4–6 kHz) exhibited the most consistent association with postoperative speech recognition among the variables analyzed. Across both BCI and AMEI users, BC-High thresholds demonstrated a stronger relationship with postoperative SRS than low- or mid-frequency BC measures, global PTA, or disease-related variables. This pattern was consistently observed across correlation analyses, multivariable regression models, and ROC analyses, highlighting the relevance of high-frequency cochlear reserve as a factor associated with postoperative functional outcomes.

Although the correlations observed between BC-High and postoperative speech recognition were statistically significant, their magnitude should be interpreted as moderate rather than strong. Correlation coefficients around 0.4 indicate that high-frequency cochlear reserve represents an important determinant of postoperative performance but does not fully account for the variability in speech outcomes. This is expected given the multifactorial nature of speech perception, which is influenced not only by cochlear sensitivity but also by neural encoding fidelity, central auditory processing, cognitive factors, the air–bone gap (ABG) and device-specific transmission characteristics.

Importantly, while BC thresholds are routinely incorporated into candidacy algorithms for implantable devices [17,18], they are traditionally used as binary eligibility criteria rather than as continuous, frequency-dependent predictors of postoperative performance. Our results extend this framework by demonstrating that high-frequency cochlear reserve appears to be importantly associated with the level of postoperative speech recognition achievable after implantation. This distinction between eligibility and outcome prediction is clinically relevant and has not been systematically addressed in comparative analyses of BCIs and AMEIs.

The predominance of BC-High over mid- and low-frequency measures is physiologically coherent and strongly supported by contemporary auditory science [21,22,23]. Speech recognition—particularly open-set word identification at conversational levels—depends especially on access to high-frequency spectral cues, which encode consonantal contrasts, fricative energy, and rapid spectral transitions essential for lexical disambiguation [24,25]. Although frequencies below 2 kHz dominate vowel perception and overall loudness, they contribute comparatively little to intelligibility once basic audibility is ensured [26].

On the other hand, BC-High thresholds reflect the integrity of the basal cochlear region, where early sensorineural degeneration, synaptopathy, and loss of frequency selectivity typically emerge [27]. Experimental and clinical evidence has repeatedly shown that even subtle deterioration in extended or high-frequency hearing—often undetected by conventional PTA—correlates with poorer speech recognition in challenging listening conditions, including speech-in-noise and multitalker environments [27,28]. Importantly, this relationship persists even in individuals classified as normally hearing by standard audiometry, highlighting the limitations of PTA-based predictors for functional outcomes [26].

Within this framework, our finding that BC-Mid (1–2 kHz) and BC-Low (250–500 Hz) showed no meaningful association with postoperative SRS is not paradoxical but expected. Mid-frequency thresholds often remain relatively preserved in mixed and conductive hearing loss and show limited variability within implant candidate populations, reducing their discriminative power. Low-frequency BC predominantly reflects apical cochlear function, which—while relevant for loudness perception—plays a minor role in consonant discrimination and lexical access. As a result, global PTA measures, heavily weighted toward low and mid frequencies, fail to capture the cochlear constraints that ultimately limit speech recognition performance. The additional contribution of the cochlear frequency gradient (BC Slope) further supports this interpretation. A steep gradient, reflecting disproportionate high-frequency deterioration despite preserved low-frequency thresholds, identifies patients with apparently favorable PTA profiles but limited speech-processing capacity. The stronger association between BC Slope and postoperative SRS observed, especially in AMEI users, suggests that not only the absolute level of high-frequency cochlear reserve, but also its distribution across the frequency spectrum, is clinically relevant when predicting functional outcomes. It should be noted that a mild postoperative worsening of bone conduction thresholds was observed in Table 2, although without statistical significance. This finding should not be interpreted as implant-related cochlear damage, but rather as a consequence of the underlying disease context. Most cases showing BC deterioration occurred in patients with chronic otitis media, the predominant etiology in our cohort, where inflammatory activity, repeated surgery, and labyrinthine irritation may promote subtle labyrinthization phenomena.

Although BC-High emerged as a shared determinant of postoperative speech recognition across implant strategies, its functional implications differed between BCIs and AMEIs. The higher BC-High cutoff identified for AMEIs should therefore not be interpreted as contradictory but as physiologically and clinically coherent. In our cohort, AMEI recipients presented a substantially higher proportion of mixed and sensorineural hearing loss, whereas BCI users predominantly exhibited conductive profiles. Accordingly, as shown in Table 2 and Figure 2, AMEI users demonstrated poorer high-frequency AC and BC thresholds at baseline, reflecting greater basal cochlear involvement. This distribution also reflects the etiological heterogeneity typical of implantable hearing rehabilitation, including chronic otitis media, cholesteatoma, congenital malformations, stenosis, and age-related hearing loss. Importantly, etiology was included in the multivariable models and did not demonstrate an independent association with postoperative speech recognition outcomes, indicating that the relationship observed between BC-High and functional performance was not driven by a specific disease subgroup.

Within this context, AMEIs demand sufficient basal cochlear integrity to effectively convert mechanical vibration into precise neural encoding. Once this reserve is exceeded, amplification alone cannot compensate for impaired frequency selectivity, reduced temporal resolution, and degraded neural synchrony, resulting in an abrupt decline in speech recognition performance [29,30]. This mechanism explains both the steeper ROC profile and the higher BC-High cutoff observed in AMEI users. The independent negative effect of age identified in the AMEI multivariable model shown in Table 3 further supports this interpretation, as age-related cochlear and central auditory decline are likely to exacerbate the functional consequences of high-frequency cochlear damage when stimulation is unilateral and strongly cochlea-dependent. However, it is important to note that the cutoff values of the ROC curves should not be interpreted as definitive clinical thresholds. Because they were derived from the same dataset and were not externally validated, they should be considered exploratory indicators of risk rather than validated decision limits.

An important implication of these findings is the distinction between audiometric eligibility and functional prognosis. Current indication criteria for BCIs and AMEIs mainly rely on bone conduction thresholds as binary cutoffs to determine candidacy. However, this approach does not account for the variability in postoperative speech outcomes observed among patients who meet these criteria. Our results suggest that high-frequency cochlear reserve could modulate the functional ceiling of speech recognition, even within accepted indication ranges, and should therefore be considered as a prognostic marker rather than an exclusion criterion. In parallel, the ABG has long been recognized as a key element in patient selection for implantable hearing rehabilitation [31,32]. Larger ABGs generally favor bone conduction-based solutions, as these devices bypass middle ear dysfunction while preserving cochlear function. Consequently, ABG magnitude helps guide the choice between reconstructive middle ear surgery, bone conduction implants, and active middle ear implants. Our findings complement this principle by suggesting that, beyond the conductive component reflected by the ABG, high-frequency cochlear reserve represents an additional determinant of postoperative speech recognition outcomes.

From a practical perspective, incorporating BC-High into the preoperative evaluation provides prognostic information that is not captured by conventional BC metrics. Previous studies comparing BCIs and AMEIs in postoperative or refractory COM populations have mainly focused on aided thresholds, functional gain, or sound pressure requirements, identifying mid-frequency BC—particularly around 1 kHz—as a predictor of aided audibility rather than speech performance [32]. While these parameters are relevant for assessing device efficiency and audibility restoration, they do not directly address the variability in postoperative speech recognition observed among patients meeting identical candidacy criteria. Our findings extend this framework by showing that high-frequency cochlear reserve is more closely linked to suprathreshold speech outcomes, particularly when speech intelligibility, rather than audibility, is the primary clinical goal.

In this context, BC-High functions as a complementary prognostic marker rather than an alternative indication criterion. As shown in our multivariable model as well as in ROC curves, patients who fulfill candidacy thresholds but present with marked BC-High deterioration are at increased risk of limited SRS benefit, even when tonal gain is substantial. This observation helps explain why previous studies reported favorable aided thresholds without consistent improvement in peak word recognition scores, especially in mixed hearing loss populations [33,34,35]. In all scenarios, BC-High should be integrated with anatomical, clinical, and patient-specific factors to refine counseling and individualized decision-making, rather than used as an isolated determinant.

### Limitations

Several limitations should be acknowledged. First, the retrospective single-center design may limit the generalizability of the findings, although the study period and balanced representation of implant types support the internal consistency of the analyses. Second, speech recognition at 65 dB SPL in quiet conditions was used as the primary functional outcome. While this measure is widely used in routine clinical practice, it does not capture performance in noise or other real-world listening conditions, where high-frequency cochlear integrity may play an even greater role.

In addition, the BC-High cutoff values were derived from the same dataset and without external validation; therefore, they should be interpreted as exploratory risk markers rather than definitive prognostic thresholds. Although 6 kHz bone conduction measurements may show greater variability and are not universally included in standard audiometric protocols, in our institution this frequency is routinely measured as part of extended audiometric assessment. To mitigate potential variability, BC-High was defined as the mean of 4 and 6 kHz rather than relying on a single high-frequency threshold. Finally, the multivariable model intentionally included a limited number of covariates to maintain stability relative to the sample size, meaning that additional clinical variables—such as device model, device programming parameters and fitting strategies, coupling configuration, or previous surgical history—were not fully adjusted for and may still influence postoperative outcomes.

## 5. Conclusions

This study indicates that BC-High thresholds are consistently associated with postoperative speech recognition following implantable hearing rehabilitation. Among the audiological variables analyzed, BC-High demonstrated a stronger relationship with postoperative outcomes than global PTA or low- and mid-frequency BC measures. In both bone conduction and active middle ear implant users, higher BC-High values were associated with poorer postoperative speech recognition, and the exploratory implant-specific cutoffs observed likely reflect differences in baseline cochlear reserve between groups rather than device-related effects. These findings suggest that evaluating high-frequency cochlear reserve may provide useful complementary information for preoperative counseling and individualized treatment planning within current indication frameworks.

## Figures and Tables

**Table 1:** Demographic characteristics, clinical variables, and etiological distribution of the study population, BCI and AMEI. * *p* < 0.05.

Variables	Overall			BCIs			AMEIs			*p* Value
**Gender (Men/Women)**	48 (47.06%)		54 (52.94%)	28 (56.00%)		22 (44.00%)	21 (40.38%)		31 (59.62%)	0.110
**Age at implantation**	57.26 ± 19.90 years			49.46 ± 20.76 years			64.77 ± 15.90 years			0.002 *
**Ear (Right/Left/Both)**	46.08%	47.06%	6.86%	40.00%	48.00%	12.00%	50.00%	48.08%	1.92%	0.105
**Hearing loss evolution**	13.54 ± 10.48 years			13.46 ± 14.27 years			13.62 ± 4.61 years			0.912
**Type of hearing loss**	Conductive		23 (22.55%)	Conductive		20 (40.00%)	Conductive		3 (5.77%)	<0.001 *
	Mixed		73 (71.57%)	Mixed		30 (60.00%)	Mixed		43 (82.69%)	
	SNHL		6 (5.88%)	SNHL		0 (0.00%)	SNHL		6 (11.54%)	
**Type of implant**	BCI		50 (49.01%)	Percutaneous Transcutaneous		28 (56.00%) 22 (44.00%)	VSB		52 (100%)	-
	AMEI		52 (50.98%)							
**Etiology**	COM		36 (35.29%)	COM		27 (54.00%)	COM		9 (17.31%)	<0.001 *
	Cholesteatoma		42 (41.18%)	Cholesteatoma		17 (34.00%)	Cholesteatoma		25 (48.07%)	
	Stenosis		8 (7.84%)	Stenosis		2 (4.00%)	Stenosis		6 (11.54%)	
	Malformation		8 (7.84%)	Malformation		4 (8.00%)	Malformation		4 (7.69%)	
	Genetic		2 (1.96%)	Genetic		0 (0.00%)	Genetic		2 (3.85%)	
	Otosclerosis		3 (2.94%)	Otosclerosis		0 (0.00%)	Otosclerosis		3 (5.77%)	
	Presbycusis		3 (2.94%)	Presbycusis		0 (0.00%)	Presbycusis		3 (5.77%)	

**Table 2:** Pre-implant and post-implant audiological outcomes in patients treated with BCI and AMEI.

Frequencies/Situation		Pre-Implant (dB/%)		Post-Implant (dB/%)		Gain (dB/%)	
**BCIs**							
**AC 250 Hz**	**BC 250 Hz**	65.79 ± 21.65	27.66 ± 24.88	41.41 ± 17.69	29.12 ± 22.06	24.38 ± 28.09	−1.46 ± 33.25
**AC 500 Hz**	**BC 500 Hz**	69.45 ± 19.77	29.89 ± 17.30	34.23 ± 17.26	33.07 ± 25.54	35.22 ± 26.26	−3.18 ± 30.85
**AC 1000 Hz**	**BC 1000 Hz**	69.55 ± 20.70	32.28 ± 18.37	30.92 ± 13.14	38.02 ± 22.12	38.63 ± 24.52	−5.74 ± 28.75
**AC 2000 Hz**	**BC 2000 Hz**	68.04 ± 21.43	36.47 ± 18.77	36.58 ± 14.80	37.11 ± 17.33	31.46 ± 26.04	−0.64 ± 25.55
**AC 4000 Hz**	**BC 4000 Hz**	74.77 ± 19.18	35.68 ± 20.24	40.92 ± 17.85	36.88 ± 18.69	33.85 ± 26.15	−1.20 ± 27.55
**AC 6000 Hz**	**BC 6000 Hz**	78.64 ± 23.19	34.31 ± 22.03	39.87 ± 17.95	34.94 ± 23.76	38.77 ± 29.30	−0.63 ± 32.40
**Mean PTA (dB)**		72.12 ± 22.08		39.07 ± 26.39		33.05 ± 34.41	
**SRS (%) at 65 dB**		12.19 ± 20.39		74.21 ± 27.01		62.02 ± 33.84	
**AMEIs**							
**AC 250 Hz**	**BC 250 Hz**	65.91 ± 18.35	41.93 ± 29.90	34.61 ± 17.86	43.54 ± 26.88	31.30 ± 25.61	−1.61 ± 30.22
**AC 500 Hz**	**BC 500 Hz**	66.59 ± 20.74	36.45 ± 15.04	33.02 ± 11.16	39.02 ± 19.02	33.57 ± 23.55	−2.57 ± 24.19
**AC 1000 Hz**	**BC 1000 Hz**	69.44 ± 22.39	40.00 ± 14.87	32.18 ± 11.50	42.16 ± 17.16	37.26 ± 25.17	−2.16 ± 22.71
**AC 2000 Hz**	**BC 2000 Hz**	73.95 ± 21.88	47.65 ± 15.01	34.20 ± 11.46	51.88 ± 19.25	39.75 ± 24.70	−4.23 ± 24.38
**AC 4000 Hz**	**BC 4000 Hz**	77.69 ± 20.73	49.11 ± 15.20	39.61 ± 13.67	54.33 ± 18.44	38.08 ± 24.83	−5.22 ± 23.88
**AC 6000 Hz**	**BC 6000 Hz**	86.35 ± 19.56	52.25 ± 18.40	41.03 ± 15.35	55.67 ± 21.36	45.32 ± 24.86	−3.42 ± 28.16
**Mean PTA (dB)**		73.91 ± 20.44		35.65 ± 9.38		38.26 ± 22.50	
**SRS (%) at 65 dB**		11.84 ± 19.34		85.79 ± 21.82		73.95 ± 29.14	

**Table 3:** Spearman correlation analysis between frequency-segmented preoperative BC measures and postoperative SRS stratified by implant type (BCI and AMEI). * *p* < 0.05.

Variable		BCIs	AMEIs
**BC-Low (250–500 Hz)**	r value (CI 95%)	−0.092 (−0.274 to 0.078)	−0.046 (−0.234 to 0.141)
	*p* value	0.2691	0.619
**BC-Mid (1000–2000 Hz)**	r value (CI 95%)	−0.081 (−0.289 to 0.126)	−0.108 (−0.284 to 0.067)
	*p* value	0.434	0.221
**BC-High (4000–6000 Hz)**	r value (CI 95%)	−0.382 (−0.566 to −0.197)	−0.398 (−0.571 to −0.224)
	*p* value	0.001 *	<0.001 *
**BC Slope (High–Low)**	r value (CI 95%)	−0.329 (−0.558 to −0.052)	−0.503 (−0.692 to −0.251)
	*p* value	0.017 *	0.002 *

**Table 4:** Results of the multivariable model including the independent variables associated with postoperative functional auditory outcomes (SRS) after implantation. * *p* < 0.05.

Independent Variables		Bone Conduction Implants	Active Middle Ear Implants
**BC-Low (250–500 Hz)**	β coefficient	−0.352	−0.214
	CI (95%)	−0.796 to 0.092	−0.514 to 0.086
	*p* value	0.118	0.158
**BC-Mid (1000–2000 Hz)**	β coefficient	−0.287	−0.263
	CI (95%)	−0.679 to 0.106	−0.603 to 0.077
	*p* value	0.149	0.126
**BC-High (4000–6000 Hz)**	β coefficient	−0.533	−0.491
	CI (95%)	−0.983 to −0.082	−0.901 to −0.081
	*p* value	0.022 *	0.020 *
**Age (Years)**	β coefficient	0.013	−0.454
	CI (95%)	−0.336 to 0.363	−0.869 to −0.039
	*p* value	0.938	0.033 *
**Disease evolution (Years)**	β coefficient	−0.040	0.724
	CI (95%)	−0.566 to 0.486	−0.963 to 2.411
	*p* value	0.879	0.391
**Etiology**	β coefficient	0.542	5.113
	CI (95%)	10.606 to 11.690	−6.583 to 16.810
	*p* value	0.922	0.382
**Surgical technique**	β coefficient	1.713	−5.115
	CI (95%)	−13.305 to 16.731	−19.152 to 8.923
	*p* value	0.819	0.466

## Data Availability

The original contributions presented in this study are included in the article. Further inquiries can be directed to the corresponding author.
